# Book Reviews

**Published:** 1799-12

**Authors:** 


### NATURAL HISTORY.

*The Britiſh Flora, or a Linnæan Arrangement of Britiſh Plants; with their generic and ſpecific characters, ſelect ſynonyms, Engliſh names, places of growth, duration, times of flowering, and references to figures. By* JOHN HULL, M. D. &c. in two parts, 8vo. 449 pp. (price nine ſhillings) *Mancheſter*, CLARKE; *London*, BICKERSTAFF; and *Edinburgh*, MUDIE.

This compendious and uſeful work has hitherto eſcaped our attention, though it was publiſhed in the beginning of the preſent year. The author appears to have taken uncommon pains, in rendering this publication of ſuperior utility to others that have hitherto appeared on the native plants of this iſland; as he has not only availed himſelf of the beſt authorities, but likewiſe added many original remarks and obſervations made in his botanical excurſions.

In order to enable the reader to judge, in ſome degree, of the merits claimed by this "*Flora*," we ſhall quote the author's own words from his preface: "The numerous corrections and additions, which the catalogue of Britiſh plants has lately received, in conſequence of the prevailing taſte for botanical purſuits, having rendered the Enchiridion Botanicum of Broughton, and all ſimilar publications, very inſufficient for the purpoſes of the practical botaniſt, the author was induced, in the beginning of laſt year, to prepare the volume, now offered to the public, for the preſs.

"Had he then been appriſed, that a pocket Flora would ſo ſoon appear by another hand, he would have relinquiſhed his undertaking; but, having nearly completed the work, before the Synopſis of Mr. SYMONS was announced for publication; and finding that work written in the Latin language, leſs comprehenſive in its plan, and not continued through the three laſt orders of the Claſs Cryptogamia, he could not conſider it as ſuperſeding the neceſſity of this volume; which, he flatters himſelf, will prove an uſeful companion in botanical excurſions.

"In the *Catalogue* here given, will be found all the *ſpecies* contained in the third edition of Dr. WITHERING's very valuable work on the botany of theſe kingdoms, together with ſuch additional ones, as have been ſince diſcovered and aſcertained. All the varieties alſo are given, except thoſe depending merely upon ſize or colour.

"In the *Arrangement*, the author has adhered ſtrictly to the method eſtabliſhed by the juſtly celebrated Linnæus, in preference to the reformed ſyſtem of Profeſſor THUNBERG; although this has been adopted on the Continent by HAENKE, in his edition of the Genera Plantarum, and by WILDENOW in his Floræ Berolinenſis Prodromus; and in this kingdom, by SIBTHORPE, WITHERING, and SYMONS. That he may not unneceſſarily add to the ſize of this volume, he will aſſign his reaſons for this, and give ſome obſervations on the botanical language, here employed, in the the preface to a work, containing an Introduction to Botany, and the Natural Characters of the Genera of British Plants, which is now in the press; and will content himself with giving here the following short statement of the plan of the British Flora.

"The *Genera* are numbered in the same manner, as in RICHARDSON's edition of the Genera Plantarum, and the fourteenth edition of the Systema Vegetabilium, published by MURRAY, at Goettingen, in 1784. The *Characters* are, in general, translated from the works of LINNÆUS, and chiefly from the Systema Vegetabilium. Where Linnæan characters are wanting, the deficiency has been supplied from the works of HUDSON, LIGHTFOOT, WITHERING, DIXON, SMITH, the Linnæan Transactions, &c. &c. With these, are occasionally given some additional distinctive marks, either included in a parenthesis, or subjoined as an observation.

"To each *Species* are added,

1. The English Names; except in the three last orders of the class Cryptogamia, where they have been almost universally omitted, because they are mere translations, and not properly established:

2. The general *Habitation*, or *Situation*, in which it is found, and, in some instances, where the plant is very rare, the particular place is indicated:

3. The *Duration*; which is expressed by the initials of the words annual, biennial, perennial, shrub, tree:

4. The *Seasons*, or *months of flowering*; the months being expressed by numbers, e. g. January by 1, February by 2, &c. &c.

5. A *Reference* to some figure, or figures. In general, one figure only is referred to, and, in some instances, an inferior English figure has been preferred to a superior foreign one, as being more accessible to the generality of readers. When two or more are given, the first place has not always been assigned to the best.

"The *Synonyms* of HUDSON, LIGHTFOOT, and WITHERING, are also constantly added, when they differ from the Linnæan name, or from each other. And the synonyms of other authors are frequently given, especially in the class Cryptogamia.

To such species as are *doubtful natives*, a note of interrogation is affixed."

---

*Botanisches Taschenbuch für die Anfänger dieser Wissenschaft, und der Apotheker-kunst, auf das Iahr*, 1799.—A Botanical Pocket-Book, for the use of the student of this science, as well as of Pharmacy, for the year 1799. By D. H. HOPPE, 8vo. 225 pp. (price 21 grosch. or about 3s. 6d.) *Regensburg*, Montag and Weiss.

Germany cannot boast of a more popular and useful botanical almanack than the present, which has now been continued for a whole *decennium*, with undiminished reputation. Its contents are well adapted to promote the study of botany; a science which is more than any other entitled to universal attention, and in which we may reasonably expect a beneficial reform in the present revolutionary age. By such a reform, however, we do not mean to insinuate, that the systematic arrangement or classification of plants, can be much altered and improved; nor is it from new discoveries of rare and curious plants, that the world will be benefitted in a degree adequate to these pursuits: but but we fubmit to the confideration of the reader, whether the *ufeful and œconomical purpofes of plants* are fufficiently inveftigated, underftood, or applied?* Our anfwer cannot be given in the affirmative.

The firft article of this 'Pocket-Book,' is a Journal kept in Spring 1798, to record the time of flowering of feveral vernal plants; by I. N. GEBHARD. The obfervations were made at Süttfchlag, fituated in a narrow valley extending from north to fouth, to the chain of mountains called Tauern, in the Bifhoprick of Salzburg, in Germany, and expofed to a rough climate, where the winter begins early in autumn, and the fnow melts only after the fpring feafon has confiderably advanced. On thefe German Alps, the author found the *Gentiana verna* in bloffom, on the 1ft of March; the *Saxifraga oppofitifolia*, which confifted here uniformly of five, and fometimes of fix petals, on the 10th of March; the *Ranunculus nivalis*, on the 17th; the *Viola biflora*, generally with *one* flower, on the 24th; the *Arnica Bellidiaftrum*, and *Cardamine bellidifolia*, on the 27th; the *Soldanella alpina*, on the 30th; the *Valeriana tripteris*, on the 4th of May; the *Ribes alpinum*, and *Gentiana acaulis*, on the 7th; the *Anemone alpina* and *vernalis*, *Pinguicula alpina, and Salix arfbufcula*, on the 9th; the *Arabis bellidifolia*, *Antirrhinum alpinum*, on the 10th; the *Bartfia alpina*, *Rhododendron ferrugineum*, *Saxifraga ftellaris*, *Potentilla aurea*, and *Azalea procumbens*, on the 14th; the *Saxifraga Cotyledon*, *Myagrum faxatile*, *Hieraceum aureum*, *Campanula barbata*, *Gypfophila repens*, and *Thymus Alpinus*, on the 19th; the *Eryfimum fulphureum*, on the 22d; the *Lonicera alpigena*, and *Senecio alpinus*, on the 28th; the *Tuffilago alpina*, *Carduus defloratus*, *Atragene alpina*, *Veronica aphylla* and *integrifolia*, *Uvularia amplexifolia*, *Ranunculus aconitifolius*, *Saxifraga rotundifolia*, *Silene acaulis*, *Sedum rubens*, *Pedicularis comofa and recutita*, *Ciftus celandicus*, *Primula minima*, and *Empetrum nigrum*, on the 30th and 31ft of May.

The next article is a paper written by the fame author, and containing reflections on the arrangement of our botanical text-books: the third is a lift of fome of the Cryptogamia, growing in the mountains of Salzburg, near *Hüttfchlag*; both by the fame author.—Some accounts of the Auftrian Alps and their plants, by L. TRATTINIK, of Vienna.—A botanical excurfion to fome of the Alps of Salzburg, Carinthia, and Tyrol, by the editor. The author calculates the height of Mount Glockner, at 6400 feet above the horizon of Klagenfurt: the number of plants collected on this journey, he reckons at 6000, among which are many new and rare alpine plants, e. g. of the former, the *Soldanella alpina*, *Atragene alpina*, &c. *Ranunculus nivalis*, *Gentiana nivalis*, *Arnica glacialis* *Artemifia glacialis*, and *Rannunculus glacialis*: of the latter defcription are the new fpecies of the *Hieracium piliferum* and *anguftifolium*, and quite a new fpecies, *Cynofurus ovatus*.

The other papers of this annual work are to us lefs interefting, as they are of a local nature; but we cannot, in this place, omit to mention a work of Mr. HOPPE, the prefent editor, entitled, *Herbarium vivum plantarum rariorum, præfertim alpinarum*. It is publifhed in numbers, each containing one hundred plants preferved in fuch perfection, as

* See an account of a new work on this fubject, in our prefent Number, under the head of *Domeftic Intelligence*, p. 482.

as is unrivalled by any similar attempt: to each plant is affixed a printed paper, with its systematic name and technical definition; the general and particular habitation of the plant; the day on which it was found; and the name of the botanist who collected and communicated the plant to the editor.

---

*Ericorum Icones et Descriptiones, auctore* JOHANNO CHRISTOPHORO WENDLAND. Fascicul. III. *Abbildung und Beschreibung der Heiden.* The three first Numbers, 4to. 1798 and 1799, (price seven rix-doll. or about 1l. 8s.) *Hanover*, HAHN.

This work was begun by the editor in conjunction with Dr. RŒMER, in 1794, who has since relinquished the undertaking, on account of adventitious circumstances. Mr. WENDLAND being the director of the Royal Garden at Herrnhausen, near Hanover, is happily situated, and eminently qualified to communicate to his countrymen the different species of heaths, partly cultivated in that magnificent botanic garden, and partly represented and described in splendid, but expensive English works. Each number contains six plates, on each a different species, not numbered, in order to arrange them at pleasure, when the whole is concluded. The drawings are indeed remarkably correct, but the colouring is inferior to most botanical plates we have lately seen imported from Germany.

The usual description of the character of every species is added with punctual fidelity, and the following are given in these three numbers: *lutea, ramentacea, Pattersoniana, pinea Thunbergii, perspicua, pinea Plukenetii, taxifolia, strigosa, spicata, interrupta Plukenetii, Bergiana, laniflora, coccinea, ventricosa, nutans, incarnata, capitata,* and *curviflora.*

---

*Beschreibung merkwürdiger Höhlen.*—A Description of remarkable Caverns; a Contribution to the Physical History of the Earth. By Dr. ROSEMULLER and Dr. TILLESIUS, 8vo. XVI. and 224 pp. with ten plates, (price three rix-doll. or 12s.) *Leipzig*, Breitkopf and Hartel.

Our room does not permit us to mention even the names of that multitude of original works from which this interesting description has been collected; but, to gratify the curiosity of the reader, we shall give an account of the different caverns here described: viz. the Devil's Arse, and Elden Cave, near Castleton; Pool's Cave, near Buxton, in Derbyshire; the Cavern near Kiemel's House, in Wales; Mortimer's Cave, in Nottingham; the caverns near Slains and Dunbar, in Scotland; the Caves of Sutherland, Caithness, Cantyre, and near Flamborough-Head; the Caves of the Isle of Arran in Scotland; the Cavern in the Hebridic Isle of Bay; the Cave of Fingal, on Staffa; the Caves of Angus-shire; the Baar's Cave in Suther-Island; the Caves of Sniofell and Snurth, on the Wester-Island; the Cavern in New Spain; the Cave at Dondon, on the Island of Hispaniola; the Cavern on the Copper Island; the Cole's Cave at Barbadoes; the Cave of Pustgeskoi, on the mountains of Alta; the Caverns near Murom in Russia; the Cave near Kungur, and those near the banks of the rivers Yenisei and Onon, in Siberia; the Caves near the Ulu Syr and Syokul in Siberia; the Bone-Caves in Egypt; the Cave of Sybilla on Lake Avern; the Grotta di Posilippo and Dog's Cave,

Cave, near Naples; the Grotto of Serpents, near Civita Vecchia; the Cave at Caftro Pales; the great Caverns at Alcantara, near Lifbon; the fmall yellow Cave in the valley of Alcantara, together with an account of their productions, never before defcribed; the Caves near Saffenage; the Witches Grotto, near Ganges, in the Sevennes; the Cave of Pilate, in the Swifs Alps; the Caves of Bruder Bahn and Glaris, in Switzerland; the Cavern in the Landgraviate of Saufenberg; the Caves of the Wefter-Foreft; the Dragon's Hole, in the Landgraviate of Heffe Darmftadt; the Cave near Bredewinde, in the Upper Palatinate; the Cave near Ribar, in the county of Zoll; the Ice-cave, near Scelicze, and the Lungs-Cave, near the Carpatic Mountains; the Horfe-hole, near Eifenach; and the Moufe-Cave, in the Dutchy of Coburg.

The defcription of the two Caves in the Valley of Alcantara, is mafterly and original; but the plates, though engraved by the editors themfelves, are, except one or two, very indifferent performances. In fhort, it appears doubtful to us, whether works of this nature afford to the mineralogift, or rather geologift, that degree of inftruction which he expects from their perufal; or whether they are not better calculated to amufe the fuperficial inquirer, as well as to infpire the admirer of Nature with dignified conceptions, than to anfwer the practical purpofes of fcience, and its ufeful application.

